# Supplementary material for: Risk of Absence of Measles Antibody in Healthcare Personnel and Efficacy of Booster Vaccination
Source: Vaccines (Basel). 2021 May 12;9(5):501. doi: 10.3390/vaccines9050501 (PMC8151488; doi:10.3390/vaccines9050501)
Supplement: Supplementary file 1 [file vaccines-09-00501-s001.zip › vaccines-1196289-supplementary.pdf]

vaccines

MDPI

Table S1. Seropositivity according to sex and birth year

Sex	Birth year	mIgG-Ab negative/equivocal *N* = 234	mIgG-Ab positive *N* = 2651	*P*-value
Female				
	<1970s	4 (2.0%)	212 (10.3%)	
	1971-1980	14 (6.9%)	425 (20.7%)	
	1981-1984	15 (7.4%)	233 (11.4%)	
	1985-1990	23 (11.4%)	465 (22.7%)	<0.001*
	1991-1994	90 (44.6%)	479 (23.4%)	
	≥ 1995	56 (27.7%)	237 (11.6%)	
Male				
	<1970s	0 (0%)	201 (33.5%)	
	1971-1980	5 (15.6%)	111 (18.5%)	
	1981-1984	2 (6.3%)	49 (8.2%)	
	1985-1990	7 (21.9%)	103 (17.2%)	<0.001*
	1991-1994	14 (43.8%)	104 (17.3%)	
	≥ 1995	4 (12.5%)	32 (5.3%)	

* χ^2 test

mIgG-Ab: measles Immunoglobulin G Antibody

Table S2. Seropositivity according to previous vaccination history and birth year

Previous history of vaccination		mIgG-Ab		Total	P-value
**Birth year**	**Since last vaccination**	**negative/ equivocal**	**Positive**		
Recorded history of vaccination: None					
<1970s		3	399	402	<0.001*
1971-1980		19	504	523	
1981-1984		16	242	258	
1985-1990		10	95	105	
1991-1994		30	93	123	
≥ 1995		20	11	31	
Recorded history of vaccination: once					
<1970s	0-10 years	1	13	14	N.A.
	Over 11 years	0	0	0	
1971-1980	0-10 years	0	32	32	N.A
	Over 11 years	0	0	0	
1981-1984	0-10 years	1	35	36	0.866†
	Over 11 years	0	1	1	
1985-1990	0-10 years	1	28	29	0.792†
	Over 11 years	18	383	401	
1991-1994	0-10 years	2	59	61	0.008†
	Over 11 years	65	343	408	
≥1995	0-10 years	3	45	48	0.084†
	Over 11 years	33	175	208	
Recorded history of vaccination: twice or more					
<1970s	0-10 years	0	1	1	N.A
	Over 11 years	0	0	0	
1971-1980	0-10 years	0	0	0	N.A

	Over 11 years	0	0	0	
1981-1984	0-10 years	0	4	0	N.A
	Over 11 years	0	0	0	
1985-1990	0-10 years	1	59	60	0.822†
	Over 11 years	0	3	3	
1991-1994	0-10 years	6	75	81	0.972†
	Over 11 years	1	13	14	
≥ 1995	0-10 years	1	27	28	0.063†
	Over 11 years	3	11	14	

* χ^2 test, † Fisher's exact test

mIgG-Ab: measles Immunoglobulin G Antibody; N.A: not applicable
